# Applications of High and Ultra High Pressure Homogenization for Food Safety

**DOI:** 10.3389/fmicb.2016.01132

**Published:** 2016-08-03

**Authors:** Francesca Patrignani, Rosalba Lanciotti

**Affiliations:** Department of Agricultural and Food Sciences, University of BolognaBologna, Italy

**Keywords:** high pressure homogenization, ultra high pressure homogenization, pathogenic species, inactivation, food matrices, non-thermal technologies, spores, enzymes

## Abstract

Traditionally, the shelf-life and safety of foods have been achieved by thermal processing. Low temperature long time and high temperature short time treatments are the most commonly used hurdles for the pasteurization of fluid foods and raw materials. However, the thermal treatments can reduce the product quality and freshness. Consequently, some non-thermal pasteurization process have been proposed during the last decades, including high hydrostatic pressure, pulsed electric field, ultrasound (US), and high pressure homogenization (HPH). This last technique has been demonstrated to have a great potential to provide “fresh-like” products with prolonged shelf-life. Moreover, the recent developments in high-pressure-homogenization technology and the design of new homogenization valves able to withstand pressures up to 350–400 MPa have opened new opportunities to homogenization processing in the food industries and, consequently, permitted the development of new products differentiated from traditional ones by sensory and structural characteristics or functional properties. For this, this review deals with the principal mechanisms of action of HPH against microorganisms of food concern in relation to the adopted homogenizer and process parameters. In addition, the effects of homogenization on foodborne pathogenic species inactivation in relation to the food matrix and food chemico-physical and process variables will be reviewed. Also the combined use of this alternative technology with other non-thermal technologies will be considered.

## Introduction

For several decades, the food research has been focused on finding new solutions to old problems to ensure food safety without losing nutrients and more recently, in response to market demand, to preserve the sensory and the freshness of the products as well as to produce innovative foods, using safe, quick, economical, and environmental friendly processes (Zamora and Guamis, 2015). In a globalized market, the solution to these goals is fundamental for the survival of food and equipment enterprises, especially the small and medium enterprises (SMEs). The enterprise competition makes crucial the innovation in processes and products and user-friendly processes have been designed, focusing the interest on the application of new processing technologies including high pressure homogenization (HPH) and ultra high pressure homogenization (UHPH; Dumay et al., 2013).

**FIGURE 1:**
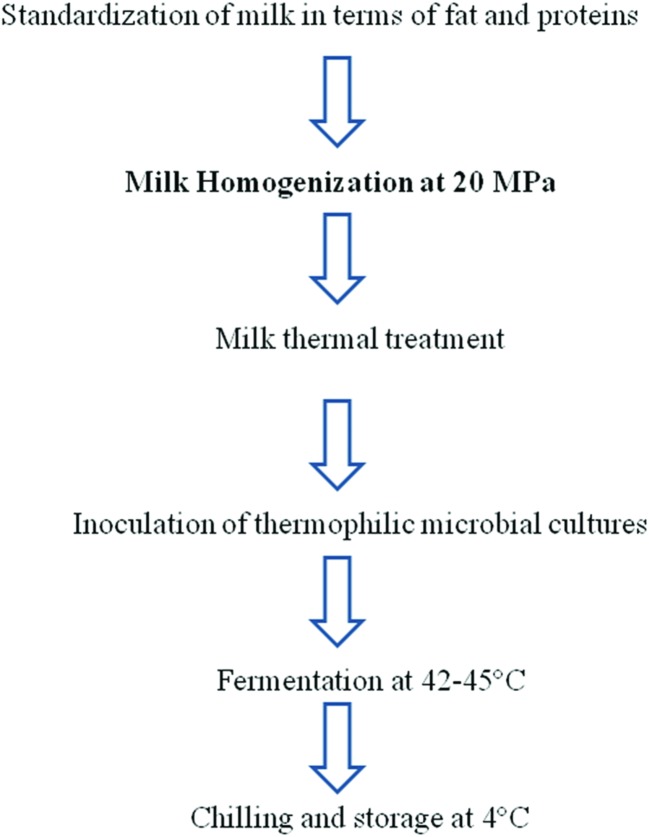
**State of art of conventional homogenization in yoghurt preparation**.

The word “homogenization” is referred to the ability to produce a homogeneous size distribution of particles suspended in a liquid, by forcing the liquid under the effect of pressure through a specifically designed homogenization valve. Homogenizer able to process fluid matrices at pressure ranging between 20–100 MPa are nowadays employed in the dairy beverage, pharmaceutical, and cosmetic industries mainly to reduce particle size and consequently increase stability of emulsions in order to avoid creaming and coalescence phenomena (**Figure 1**). However, HPH was firstly employed as an useful method for cell disruption and recovery of intracellular bio-products (Keshavarz Moore et al., 1990; Shirgaonkar et al., 1998). The successful results obtained on cell disruption of dense microbial cultures stimulated researches on the application of HPH for food safety and shelf-life improvement. In fact, in the food industry, there is a growing interest in mild non-thermal processes, which combine an efficient microbial reduction with a maximal retention of physic-chemical product properties, as well as nutritional and sensory characteristics of the raw materials and ingredients used. Among the non-thermal treatments studied and proposed, HPH is regarded by a wide literature as one of the most encouraging alternatives to traditional heat treatments for food preservation and diversification dairy products, emulsions, egg based foods and fruit juices. Its effectiveness in the deactivation of spoilage microorganisms in model and real systems is well documented since 1994 (Lanciotti et al., 1994, 1996; Guerzoni et al., 1999; Kheadr et al., 2002; Wuytack et al., 2002; Vannini et al., 2004; Diels and Michiels, 2006; Bevilacqua et al., 2009; Patrignani et al., 2009a, 2010, 2013a,b; Zhao et al., 2014; Ferragut et al., 2015). However, HPH effectiveness for microbial inactivation is affected by several parameters such as process and microbial-physiological factors and aspects related to the characteristics of the treated fluid.

The HPH has been proposed also for the bacterial spore inactivation, generally in combination with other physic-chemical hurdles for spore surviving and/or germination due to the ability of bacterial spores to survive to the most prohibitive conditions (Wuytack et al., 2002; Bevilacqua et al., 2007, 2012; Chaves-López et al., 2009; Chen et al., 2013; Roig-Sagues et al., 2015). In addition to the effects on microbial cells, the HPH treatment is reported to act on food constituents, especially proteins and enzymes, modifying their functional properties and activities (Kheadr et al., 2002; Hayes and Kelly, 2003; Vannini et al., 2004; Iucci et al., 2008; Navarro et al., 2014). In fact, it is reported to improve food microstructure, rheology and availability of food bioactive compounds (Guerzoni et al., 1997, 2002; Lanciotti et al., 2004a,b; Sandra and Dalgleish, 2005; Betoret et al., 2015). Moreover, it has been proposed as a useful tool to enhance the cheese yield and reduce the cheese ripening times due to the enhancement of proteolysis and lipolysis of milk naturally occurring or microbial enzymes (Guerzoni et al., 1999; Lanciotti et al., 2004b, 2006, 2007a; Burns et al., 2008; Vannini et al., 2008; Burns et al., 2015). Moreover, the effects of HPH, applied at sub-lethal level (50 MPa), was studied on several strains of *Lactobacillus* spp., and particularly non-starter LAB (NSLAB), inoculated in milk as adjunct and involved in dairy product ripening (Lanciotti et al., 2007b). The literature data demonstrated that this cold technology is able to affect the strain metabolic activity and enzymes leading to a modification of the cheese ripening patterns, especially linked to the breakdown of proteins. The modification of enzyme and protein activity and functionality can greatly affect not only the food quality and ripening patterns but also the food safety feature due to the increased activities of egg and milk naturally occurring antimicrobials such as lysozyme, lactoperoxidase systems, and lactoferrin (Vannini et al., 2004; Iucci et al., 2007; Patrignani et al., 2013b) or reducing the biogenic amine content in cheeses obtained from HPH treated milk (Lanciotti et al., 2007a). In addition, HPH has been proposed with several roles in the functional food sector, for the production of probiotic dairy products with improved sensory or functional properties, such as probiotic strain viability over refrigerated storage and accelerated fermentation kinetics with less environmental impact with respect to the traditional heat treatment (Burns et al., 2008, 2015; Patrignani et al., 2009b, 2015a). In fact, sub-lethal pressure levels (50 MPa) applied directly to microbial cells, increased some functional properties (i.e., hydrophobicity, resistance to simulated gastric conditions, and stomach-duodenum passage) in some probiotic strains associated to an increased maintenance of viability during refrigerated storage (Tabanelli et al., 2012, 2013, 2014). Also, Muramalla and Aryana (2011) reported low homogenization pressures (up to 13–80 MPa for five passes) to improve certain probiotic characteristics of yogurt bacteria and *Lactobacillus acidophilus* LA-K (i.e., strain acid and bile tolerance) without effects on protease activity and strain growth potential. Additionally, these low homogenization pressure treatments, applied directly to probiotic strains, modified their interaction with the small intestines of BALB mice and induced a higher IgA response compared with untreated mice in a strain- and feeding period-dependent way (Tabanelli et al., 2012). Burns et al. (2015) studied the effects of a sub-lethal HPH on a probiotic strains used as adjunct for producing Caciotta cheese demonstrating that the HPH-treated probiotic strain maintained high viability for 14 days whilst the physico-chemical analyses on Caciotta cheese containing HPH treated cells showed a faster ripening, compared to cheeses containing not HPH treated cells. In addition, these Authors demonstrated that the 50 MPa treatment increased the *L. paracasei* gastric resistance in Caciotta, maintaining high strain viability without any significant effects on IgA production in mice.

The HPH technology has shown a great impulse both at industrial level and research field during the last decades also with the support of some important EU projects. The most relevant EU projects relative to the effects of HPH on food safety can be envisaged in the EU project HighQ RTE FP6-FOOD-023140 “Innovative non-thermal processing technologies to improve the quality and safety of ready-to-eat (RTE) meals-HighQ RTE,” EU Craft project “UHPH 512626, Development and Optimisation of a Continuous Ultra High Pressure Homogenizer for Application on Milks and Vegetable Milks” and the EU project FUNENTECH 232603 “study of functionality, nutritional and safety aspects of liquid foods, liquid food preparations, and cosmetics processed by ultrahigh-pressure homogenization”. The last had the aim to reinforce transfer the HPH processing to SMEs of fluid or pumpable ingredients and foods as well as cosmetic sectors.

The research performed within national and international projects stimulated the improvement of the HPH equipments and devices and their adaptation to food processing lines. Several HPH equipments are nowadays available and some equipment producers, such as Microfluidics (USA), Bee International (USA), Avestin (Canada), Gea Niro Soavi (Italy), and APV (UK), have proposed pilot devices able to exert a pressure of 100–200 MPa, with average flow of 160 L/h at 200 MPa (Zamora and Guamis, 2015). However, Stansted Fluid Power Ltd (United Kingdom) have developed from the year 2006 pilot devices able to work up to 400 MPa giving origin to the UHPH technology applied to food sector. Also Gea Niro Soavi (Italy) developed within the project made in Italy ATENA a pilot homogenizer able to work at 400 MPa, having an average flow of 5 L/h (patent no. US-2015-0314254-A1, 11/05/2015). This prototype was used to obtained apple and tomato based formulations having a shelf-life of 30 days when stored at environmental temperature and treated at 300–400 MPa. At the expire date the levels of *Listeria monocytogenes, Escherichia coli, Staphylococcus aureus, Bacillus cereus* and *Salmonella* spp. were under the detection limits even in the samples inoculated before the HPH treatments at levels ranging between 3 and 4 log CFU/ml.

Since the safety features are prerequisite both for traditional and innovative products, this review is focused on the effects of HPH on foodborne pathogen inactivation in relation to the food matrix and food physico-chemical and process variables. Moreover, the review takes into consideration also the opportunity to inactivate the spores from pathogenic bacteria by using dynamic pressure. Also the potential of HPH to increase the activity of antimicrobial enzymes to inactivate pathogenic microorganism will be taken into consideration. Finally, the combined use of this alternative technology with other non-thermal technologies will be considered within this review.

## Mechanism of Action of High Pressure Homogenization

**FIGURE 2:**
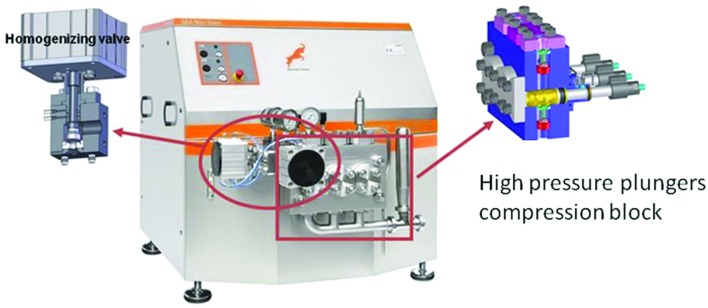
**State of the art of homogenizer able to reach 200 MPa (GEA Niro Soavi, Italy)**.

From a technological point of view, an homogenizer consists principally of a pump and a homogenizing valve (**Figure 2**). The pump is used to force the fluid into the valve where the homogenization happens (Diels and Michiels, 2006). In the homogenizing valve the fluid is forced under pressure through a small orifice between the valve and the valve seat. The operating pressure is controlled by adjusting the distance between the valve and the seat. Among the process parameters, the level of pressure applied and the temperature reached in the food matrix during the process are the variables able to affect the food components and the eventually occurring microbial cells. The microbial inactivation caused by the application of HPH, although affected by several factors and mainly by the physic chemical features of the food matrix and the sensitiveness of different microorganisms, increases with the pressure level (Diels and Michiels, 2006). The effects induced by the temperature have to be necessarily taken into account in HPH, since, during homogenization, a rise of the temperature (about 2.5°C per 10MPa), related to the fluid food employed, is observed in the fluid downstream of the valve. This is generally attributed by the literature to the viscous stresses caused by the high velocity of the fluid flow, which is then impinging on the ceramic valve of the homogenizer, leading to the dissipation of a significant fraction of the mechanical energy as heat in the fluid (Floury et al., 2000; Zamora and Guamis, 2015). However, such temperature increase did not result in the appearance of heat indicators in HPH treated food samples probably due the flash time of treatment of the food matrices (lower than 1 s; Floury et al., 2004; Pinho et al., 2011). For example HPH treated milk samples suffered less Maillard reaction, less whey protein denaturation and did not present lactose isomerisation compared to a commercial pasteurized milk with a concomitant preservation of essential amino acids and as a consequence with a better nutritional value desired by consumers (Pereda et al., 2009).

The significant improvement of HPH equipment functionality and flexibility (capacity to work in a food processing line), the design of new valves able to work at 300–400 MPa and the significant research efforts make HPH technology ready for the scaling up at industrial level for the development of new products differentiated from traditional ones by sensory and structural characteristics or functional properties. Also patents regarding continuous systems and procedure of sterilization and physical stabilization of pumpable fluids by means of HPH are available (i.e., EP 2409583 A1).

## Potential and Applications of High and Ultra High Pressure Homogenization for Pathogenic Species Inactivation *In Vitro* and Food Systems

**FIGURE 3:**
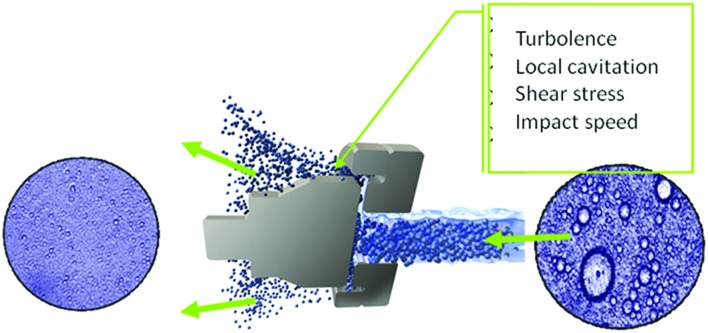
**Most probable mechanisms of action of HPH (GEA Niro Soavi, Italy)**.

The first application of homogenization for the stabilization of food and dairy emulsion was presented at the Paris World’s Fair in 1900 where Auguste Gaulin presented an invention for “intimately mixing milk” using pressures up to 30 MPa (Gaulin, 1899). Since then conventional homogenization extended the pressure range until 50 MPa. HPH, also known as dynamic HPH, has been frequently highlighted for its potential for food cold pasteurization (Diels and Michiels, 2006; Donsì et al., 2009a,b,c; Patrignani et al., 2009a, 2010, 2013a,b; Pereda et al., 2009; Panozzo et al., 2014). Modern high pressure homogenizer enables pressures 10–15 times higher than traditional ones and covers pressure ranges between 300 and 400 MPa. These last ranges have been referred to as UHPH. The progression toward UHPH has also opened the view to new sterilization opportunities, including also the inactivation of spores by HPH. The inlet temperature of pumpable products and the level of pressure, which both determine the temperature reached during the UHPH treatment, have been considered as the main factors of the microbial inactivation (Zamora and Guamis, 2015). UHPH warrants the destruction of microorganisms reaching sterilization of liquid food products, and it has a positive influence on food stability, with few effects on nutritional value and sensory characteristics of the processed fluids. UHPH technology allows more efficient particle reduction than the classical homogenization, and its effect is the results of several mechanisms such as sudden pressure drop, torsion and shear stresses, turbulence, impingement, cavitation phenomena, shock waves, and temperature increase, with a concomitant reduction of microbial load and interesting consequences in emulsion properties (**Figure 3**). Since its first appearance, HPH and, later, UHPH have been tested on several matrixes and *in vitro* systems by using different microbial targets to demonstrate their effectiveness. The microbial targets deal with pathogenic and spoilage microorganisms but in this review only the researches regarding the pathogenic species will be reviewed.

In **Table 1**, the studies regarding the microbial inactivation in model systems by HPH or UHPH, in relation to the microbial targets, inoculation level and conditions adopted, are reported. Lanciotti et al. (1994) studied the effectiveness of pressures ranging from 15 to 200 MPa, applied with a continuous homogenizer (Niro Soavi, Parma, Italy), on the cell viability of spoilage and pathogenic microorganisms in two model systems made of water 50%, oil 25%, egg yolk 25%, the first, and water 25%, oil 53%, egg yolk 22%, the second. Moreover, the effects of microstructural modifications of food systems associated with the treatments on cell viability and on its subsequent evolution were investigated. Modulation of the homogenization pressure allowed strong instantaneous reductions of the initial cell loads of *L. monocytogenes* and *Yersinia enterocolitica*. The combined effects of the initial pressure treatment and space reduction, resulting from the microstructural modifications of food systems, increased the safety and the shelf-life both of water in oil and oil in water emulsions. Moreover, Lanciotti et al. (1996) investigated also the effects of chemico-physical growth conditions such as pH, temperature and water activity *(a)* on lethal high homogenization pressure effects on *L. monocytogenes, S. aureus*, and *E. coli*. The results, based on standard medium such as Brain Heart Infusion (BHI), highlighted the importance of food system composition and its thermal history on the high pressure tolerance of the microbial population. In previous work, Lanciotti et al. (1994) showed that the relationship between survival cells and instantaneous pressure applied in medium appeared to be log-linear in a range between 40 and 200 MPa. According to the author findings, *S. aureus* was the most resistant pathogenic species while *L. monocytogenes* was resistant to pressure only when grown at highest *aw.* Also Guerzoni et al. (1997) studied the growth of *L. monocytogenes* in a model system, simulating a dairy product, when treated at 25 MPa, in relation to the lipid content NaCl and pH values, modulated according to a Central Composite Design (CCD). Polynomial equations describing the effects of such variables on the *aw*, microstructural features, and *L. monocytogenes* growth, were obtained. The three variables and their interactions, in addition to a direct effect on microbial growth, played an indirect role due to their influence on microstructural features, such as diameter of water droplets and total water phase availability. In particular, the pH value affected the a(w) and the total space available for microbial growth, while the NaCl content had a prevalently indirect effect on space availability and on the diameter of the water droplets. The results suggested that the microstructural changes induced by HPH affected the growth of *L. monocytogenes* which was dependent on the total water phase space availability. Guerzoni et al. (2002) studied the survival of *Salmonella enteritidis* after pressure treatments, ranging between 0.1 and 140 MPa, in relation to compositive variables (NaCl content, pH). The study was performed both in model (BHI) and real systems consisting of an egg-based mayonnaise type product. Moreover, the fate of the surviving cells of *S. enteritidis* was monitored during storage at 10°C and the growth or death parameters were calculated and modeled in relation to pH, NaCl concentration of the medium and the level of pressure treatment applied. From this study, it was evident that the salt content and pH displayed a synergistic effect with pressure, whose extent was higher in the mayonnaise based products than in BHI. In fact, while in the model systems the cell recovery and growth during the subsequent incubation at 10°C was allowed in many combinations of the CCD, in the real systems no recovery or growth of *S. enteritidis* were observed. According to the Authors, this viability loss, which was maximum at pH 4.00 or 2% NaCl, is not be attributed merely to the interactions of such variables, but it probably involved the naturally occurring antimicrobial enzymes of the raw material, whose activity can be enhanced by the pressure treatment. Reviewing the literature, one of the most studied *in vitro* system is represented by PBS buffer. Different Authors have investigated in this medium the potentialities of HPH treatment in pathogenic species inactivation. For example, Vachon et al. (2002) studied the effect of an homogenization treatment performed at 200 MPa, repeated for five cycles, on the inactivation of *S. enteritidis* ATCC 13047, *E. coli* O157:H7 ATCC35150 and *L. monocytogenes* LSD 105-1 when inoculated in PBS buffer at pH 7. The results showed that *E. coli* and *L. monocytogenes* reached 8 log reduction cycles after the HPH treatment after three passes while five passes were necessary to reach the same inactivation level for *S. enteritidis*. Also Wuytack et al. (2002) found that a treatment of 300 MPa was able to inactivate *S. aureus, Enterococcus faecalis* and *L. innocua* of 6, 3, and 5 log reduction cycles in PBS buffer pH 7, using a treatment of 300 MPa and a fluid inlet temperature of 25°C. Diels et al. (2003) conduced a detailed study of the inactivation of *S. aureus* and *Y. enterocolitica* in PBS buffer by HPH at, respectively, 25 and 35 different combinations of process temperature and process pressure covering a range of 5–50°C and 100–300 MPa. In the entire studied, it was clear that *S. aureus* was more resistant to HPH than *Y. enterocolitica*, as already demonstrated by Lanciotti et al. (1996). Also, temperature between 5 and 40°C did not affect inactivation of *S. aureus* by high-pressure homogenisation, while *Y. enterocolitica* inactivation was affected by temperature over a much wider range. Later, Diels et al. (2005) investigated the resistance of *E. coli* K12 when treated in PBS buffer at 300 MPa by using a axial-flow through orifice valve, outlining reduction of 6 log cycles. Donsì et al. (2009c) studied the inactivation of *E. coli* by HPH in model systems, under a wide range of operating conditions (temperature, pressure, number of homogenization passes, cell concentration) in a lab-scale and a pilot-scale unit (Stansted Fluid Power) utilizing single or multiple passes. Results highlighted that the inactivation kinetics did not depend linearly on pressure, due to the distribution of individual cell resistance in the sample. The efficacy of the treatment at higher pressures or upon multiple passes was reduced. Moreover, the scale of the apparatus, which in this case differed of several order of magnitude (from 0.7 to 120 l/h), and consequently of the width of the gap of the valve did not affect the extent of microbial inactivation at a given pressure. Taylor et al. (2007) studied the inactivation of *E. coli* K-12 cells, grown statically or in chemostat, when exposed to HPH processing pressures of 50 to 350 MPa in the absence or presence of the antimicrobial nisin. These Authors found that pressure and temperature exhibited a quadratic relationship. Significant HPH-induced inactivations of the Gram-negative microorganism was observed in the range of 100 to 250 MPa. Above 300 MPa, heat was the main factor promoting microbial inactivation, regardless of whether cells were grown in chemostat or statically. Chemostat-grown cells were significantly more resistant to HPH processing than were statically grown cells. Moreover, the data indicated a potential synergistic effects of nisin and HPH on the inactivation of bacterial contaminants, although this antimicrobial is generally active against Gram-positive bacteria.

**Table 1:** High pressure homogenization (HPH) microbial inactivation in relation to the model system, species and process conditions adopted.

Matrix	Microorganisms	Reduction	Conditions	Homogenizer/type of valve	Reference
Water 50%, oil 25%, egg yolk 25%	*Listeria monocytogenes*, *Yersinia enterocolitica*	7 log for all	Pressure ranges: 150–200 MPa	PA′NS valve	Lanciotti et al., 1994
Water 25%, oil 53%, egg yolk 22%	*Listeria monocytogenes*, *Yersinia enterocolitica*,	7 log for all	Pressure ranges: 150–200 MPa	PA′NS valve	Lanciotti et al., 1994
Brain Heart Infusion modified in pH and water activity	*Listeria monocytogenes, Staphylococcus. aureus, Escherichia coli*	7 log for all	Pressure ranges: 150–200 MPa	′PA′NS valve	Lanciotti et al., 1996
Model system simulating dairy product	*Listeria monocytogenes,*	No growth with respect to the inoculum	Pressure: 25 MPa	′PA′NS valve	Guerzoni et al., 1997
Brain Heart Infusion modified in pH and NaCl	*Salmonella enteritidis*	2.6 log	Pressure ranges: 0.1–140 MPa	PA′NS valve	Guerzoni et al., 2002
PBS buffer (pH 7)	*Staphylococcus aureus*	3.0 log	Tin = 50 *P* = 300 Tout = 18	Counterjet dispergator	Diels et al., 2003
PBS buffer (pH 7)	*Staphylococcus aureus*	6.0 log	Tin = 25 *P* = 300 Tout = 42	Counterjet dispergator	Wuytack et al., 2002
PBS buffer (pH 7)	*Yersinia enterocolitica*	6.0 log	Tin = 50 *P* = 250 Tout = 18	Counterjet dispergator	Diels et al., 2003
PBS buffer (pH 7)	*Salmonella enteritidis* ATCC 13047	8.0 log (after five passes)	Tin = 25 *P* = 200 Tout = NR, flow 1.5 L/h	Counterjet dispergator	Vachon et al., 2002
PBS buffer (pH 7)	*Enterococcus faecalis*	3.0 log	Tin = 25 *P* = 250 Tout = 42	Counterjet dispergator	Wuytack et al., 2002
PBS buffer (pH 7)	*Escherichia coli* K12	6.0 log	Tin = 25 *P* = 300 Tout = NR	Axial-flown through orifice valve	Diels et al., 2005
PBS buffer (pH 7)	*Escherichia coli* MG1655	7.0 log	Tin = 50 *P* = 250 Tout = 18	Counterjet dispergator	Diels et al., 2004
Saline solution and nisin	*Escherichia coli* K12	7.0 log	Tin = 5 *P* = 300 Tout = 70	Axial-flown through orifice valve	Taylor et al., 2007
PBS buffer (pH 7)	*Escherichia coli* O157:H7 ATCC 35150	8.0 log (after three passes)	Tin = 25 *P* = 200 Tout = NR flow 1.5 L/h	Counterjet dispergator	Vachon et al., 2002
LB nutrient	*Escherichia coli*	7.0 log	300 MPa	Stansted high-pressure homogenizer (model FPG11300:350	Donsì et al., 2009b

However, although a huge amount of data collected from model systems were available and helpful to understand the mechanisms of action of HPH against pathogenic species, many researchers tested potentialities of this technology against pathogenic species inoculated in food matrixes because the composition and viscosity of the treated food also have an indirect effect on the microbial inactivation (**Table 2**). The available literature reports about the use of HPH for the reduction of foodborne pathogens in several food matrices, such as milk (Lanciotti et al., 1994; Diels et al., 2005; Hayes et al., 2005; Brinez et al., 2006a,b, 2007; López-Pedemonte et al., 2006; Roig-Sagues et al., 2009), egg-based products (Velazquez-Estrada et al., 2008; Patrignani et al., 2013b), orange juice (Brinez et al., 2006b; Kumar et al., 2009; Pathanibul et al., 2009; Maresca et al., 2011), mayonnaise type products (Guerzoni et al., 2002). Lanciotti et al. (1994) found that the relationship between surviving cells and pressure applied was log-linear in milk inoculated with *Y. enterocolitica* and *L. monocytogenes* and processed at different pressures (40–150 MPa) at 25°C. In order to establish the fate of the surviving cells, the growth of *Y. enterocolitica*, and *L. monocytogenes*, in the samples stored at 3–4°C, was followed over 220 h. The data were analyzed according to the Gompertz equation. For *Y. enterocolitica* and *L. monocytogenes*, the treatment apparently did not induce irreversible damage to the surviving cells; in fact, although the lag phase was prolonged when pressures higher than 400 bar were used, the μmax increased with the applied pressure and the maximal cell numbers attained (A + K) were independent on the level of the applied pressure. Diels et al. (2005) studied the inactivation of *E. coli* MG1655 in skim, soy and strawberry-raspberry milk subjected to 300 MPa founding that the highest inactivation was reached in skim milk with a Tin and Tout temperature of 25 and 18°C, respectively, by using a counterjet dispergator valve. Hayes et al. (2005), utilizing an axial-flown through orifice valve, found in milk treated at 250 MPa, with a Tin of 45°C and a Tout of 76.8, a 6 log reduction of *Pseudomonas fluorescens* AFT 36, resulting from the application of pressure and temperature increase. Lowest reduction (2.7 log) was found by Brinez et al. (2006b) for *L. innocua* ATCC33090 in bovine milk or *S. aureus* ATCC 13565 (3.6 log reduction) and *S. aureus* CECT 4491 in milk (Brinez et al., 2007). López-Pedemonte et al. (2006) found high inactivation for *S. aureus* CECT 976 inoculated in milk treated at 330 MPa. Roig-Sagues et al. (2009) reported about the inactivation of *L. monocytogenes* CCUG 15526 when inoculated at 7.0log CFU/ml in milk samples having 0.3, 3.6, 10, and 15% of fat contents. The samples were subjected to a single cycle of UHPH treatment at 200, 300, and 400 MPa. Microbiological analyses were performed 2 h after the UHPH treatments and after 5, 8, and 15 days of storage at 4°C. Maximum lethality values were observed in samples treated at 400 MPa with 15 and 10% fat (7.95 and 7.46 log CFU/ml), respectively, while in skimmed and 3.6% fat milk samples, complete inactivation was not achieved and, during the subsequent 15 days of storage at 4°C, *L. monocytogenes* was able to recover. In milk samples with 10 and 15% fat, *L. monocytogenes* recovered to the level of initial counts only in the milk samples treated at 200 MPa but not in the milk samples treated at 300 and 400 MPa. According to these data, fat content increase enhanced the maximum temperature reached during UHPH treatment and this could have contributed to the lethal effect achieved. In addition the HPH treatments of milk is reported to enhance the release of free fatty acids (due to the rupture of fatty globule membranes and the activation of lipases), and mainly short and medium chain ones, having an antimicrobial effects (Lanciotti et al., 2006; Vannini et al., 2008). On the contrary, some Authors attributed to the fat content in milk a protective role against microbial species during the high pressure treatment performed at 100 MPa for several cycles. Although several Authors have tested the same matrixes, and in some case the same microbial species, different inactivation results was achieved, particularly using the multi-pass approach. For example, Patrignani et al. (2013b) found that *Salmonella* inactivation in eggs, resulting from the application of HPH at 100 MPa, seems to be linearly correlated to the number of passes. This result, although using a different substrate and microorganism, is in agreement with the findings of Patrignani et al. (2010) who demonstrated for the spoiling *Zygosaccharomyces bailii*, inoculated in apricot and carrot juice, that the effect of each pass is additive and, therefore, each homogenization pass causes almost the same reduction of the microbial load. Several other authors have also found first order inactivation kinetics as a function of the number of passes (Wuytack et al., 2002; Diels and Michiels, 2006; Tahiri et al., 2006), although the literature data concerning the inactivation kinetics by HPH are still conflicting. In fact, Donsì et al. (2009c) observed that HPH inactivation of *E. coli*, produced a non-additive trend for multiple pass processes at a given pressure level. These Authors attributed this behavior mainly to the physiological diversity within a microbial population and to the existence of resistant cells able to survive after repeated passes at the pressure applied. On the other hand, it is important to take into consideration the additional effect of HPH on the antimicrobial activity of naturally occurring enzymes, such as lysozyme, lactoperoxidase system and so on. Velazquez-Estrada et al. (2008) have published data regarding the use of HPH for *Salmonella* inactivation in liquid whole egg (LWE). In particular, these Authors proposed the single-pass treatment of inoculated LWE with ultra HPH at 100, 150, 200, and 250 MPa, demonstrating that the level of pressure applied can influence the *S. enterica* lethality attained. Moreover, Panozzo et al. (2014) outlined the potentialities of HPH as a promising alternative to thermal pasteurization of eggwhite. These Authors showed that a HPH at 150 MPa for multiple passes was able to decontaminate egg white inoculated with *S. enterica* SDMZ 9898.

**Table 2:** High pressure homogenization microbial inactivation in relation to the food matrix, species and process conditions adopted.

Matrix	Microorganisms	Reduction	Conditions	Homogenizer/type of valve	Reference
Milk	*Yersinia enterocolitica, Listeria monocytoges*	*Yersinia enterocolitica* 5 log at 150 MPa *Listeria monocytogenes* : the same	*P* range = 40–150 MPa Tout max = 65	PS valve, Gea Homogenizer	Lanciotti et al., 1994
Egg yolk 10%, yoghurt 13%, sunflower oil 60%, water in relation to pH and NaCl	*Salmonella enteritidis*	Reduction was obtained at 50 MPa with pH 4 and 2% NaCl. No re-growth at 10°C	*P* range = 0.1–50 MPa	PS valve, Gea Homogenizer	Guerzoni et al., 2002
Skim, soy, and strawberry-raspberry milk	*Escherichia coli* MG1655	Skim 3.5 log Soy 3.0 log Straw/rasp 3.0 log	Tin = 25, *P* = 300 MPa, Tout = 18	Counterjet dispergator	Diels et al., 2005
Bovine milk	*Pseudomonas fluorescens* AFT 36	6 log	Tin = 45 *P* = 250 Tout = 76.8	Axial-flown through orifice valve	Hayes et al., 2005
Milk	*Staphylococcus aureus* CECT 976	7 log	Tin = 20 *P* = 330 Tout = NR flow 16 L/h	Axial-flown through orifice valve	López-Pedemonte et al., 2006
Orange juice	*Escherichia coli* O58:H21 ATCC 10536, *Escherichia coli* O157:H7 CCUG 44857	3.9 log (O58:H21) 3.7 log (O157:H7)	Tin = 20 *P* = 300 Tout = NR, flow 18 L/h	Axial-flown through orifice valve	Brinez et al., 2006b
Milk	*Listeria innocua* ATCC 33090	2.7 log	Tin = 20, *P* = 300; Tout = NR, flow = 18 L/h	Axial-flown through orifice valve	Brinez et al., 2006a
Milk and orange juice	*Staphylococcus aureus* ATCC 13565	Milk 3.6 log Orange juice 4.2 log	Tin = 20 *P* = 300 Tout = 18 flow 18 L/h	Axial-flown through orifice valve	Brinez et al., 2007
Liquid whole egg	*Salmonella enterica* serovar *senftenberg* 775W	3.2 log at 250 MPa	100,150, 200, and 250 MPa	Not found	Velazquez-Estrada et al., 2008
Milk (0.3, 3.6, 10, and 15% fat contents)	*Listeria monocytogenes* CCUG 15526	7.95 log at 400 MPa and 15% fat	200, 300, and 400 MPa	Benchtop high-pressure homogenizer (model/DRG FPG7400H:350, Stansted Fluid	Roig-Sagues et al., 2009
Apple juice and apple cider	*Escherichia coli* K12	7 log	Tin = 25 *P* = 250 Tout = 70	Axial-flown through orifice valve	Kumar et al., 2009
Apple and carrot juice	*Listeria innocua*, *Escherichia coli*	*Escherichia coli*: > 5 log reduction (>250 MPa). *Listeria innocua*: 5 log at 350 MPa	*P* range = 0–350 MPa		Pathanibul et al., 2009
Orange, red orange, and pineapple	*Escherichia coli*	100 MPa 7 log after four passes 150 MPa 8 log after three passes	*P* range = 50–250 MPa Passes = 1–5 Tin = 2–20°C	nm-GEN 7400 series by Stansted Power Fluids, UK.	Maresca et al., 2011
Liquid whole egg	*Salmonella enteritidis*	3 log	100 MPa for five cycles	PS valve, Gea Homogenizer	Patrignani et al., 2013b
Egg white	*Salmonella enter*ica SDMZ 9898	5 log after eight passes at 150 MPa	*P* = 20, 50, 100, 150 MPa *P* = 150 MPa via multiple passes up to 17	Cylindrical tungsten carbide homogenising valves	Panozzo et al., 2014

Many applications of HPH or UHPH for inactivation of spoiling and pathogenic species were tested in juices and vegetable drinks, where HPH and UHPH find a great application potential. For example, Brinez et al. (2006a) tested the inactivation of *E. coli* 058:H21 ATCC 10536 and *E. coli* 0157:H7 CCUG 44857 inoculated in orange juice and treated at 300 MPa founding a log reduction of 3.9 and 3.7, respectively. Kumar et al. (2009) investigated on the inactivation of *E. coli* K12 in apple juice and apple cider obtaining an inactivation of 7 log applying a pressure of 250 MPa with a Tin of 25°C and Tout of 70°C, thus avoiding the use of a thermal exchanger. Also Pathanibul et al. (2009) inoculated *L. innocua* ATCC51742 and *E. coli*, as surrogate for foodborne pathogens, in apple and carrot juice, containing or not nisin (0-10 IU), and treated from 0.1 to 350 MPa. At 50 MPa homogenization pressure intervals, juice samples were collected, immediately cooled to 10°C, and then serially diluted and plated on non-selective recovery media. As processing pressure increased, inactivation of *E. coli* increased, and a 5 log reduction of cells was achieved following exposure to pressures in excess 250 MPa. In contrast, little inactivation was observed for *L. innocua* with pressures ranging between 250 and 350 MPa. However, several authors have been demonstrated the major efficacy of HPH against Gram-negative bacteria. The addition of 10 IU nisin, together with HPH, did not exhibit significant additional *E. coli* inactivation, but interactions were observed with *L. innocua.* On the other hand nisin is reported to be active against Gram-positive bacteria (Siroli et al., 2015).

## Potential and Application of High Pressure Homogenization for Spore Inactivation *In Vitro* and Food Systems

Bacterial spores represent one of the major hazard in food safety due to their high resistance to most hurdles. In particular, they are resistant to elevated temperatures (80°C), which distinguishes them from vegetative cells. Thermal sterilization is the method to eliminate spores in most food applications, as it provides the highest guarantee of sterility. From a safety point of view, spores of *Bacillus* spp. and *Clostridium* spp. are greatly resistant to several treatment such as heat, desiccation, lack of nutrients, exposure to UV and gamma radiation, organic chemicals, and oxidizing agents. In general, the heat resistance of spores depends on conditions such as elevated sporulation temperature, the presence of minerals and dipicolinic acid (DPA), and core dehydration. The endospores are composed of a central core, which is surrounded by several protective layers. The outermost layer, the exosporium, is not present in spores of all species, and is the primary site of contact with the environment. Between the outer and inner membrane, there is the cortex. Since the spore structure plays a major role in spore resistance, the spore inactivation in foods requires high levels of heat treatments, which can in turn have negative effects on the sensory and nutritional profile (Schubert and Beaudet, 2011; Reineke et al., 2013; Georget et al., 2014a,b; Dong et al., 2015). For this reason, alternative and convenient methods have been studied in recent years (Chaves-López et al., 2009). In order to increase antimicrobial effectiveness and reduce side effects on food quality, the application of combined hurdles has also received great attention. Extensive literature indicates that the effects of combined stresses on microbial growth and survival may be additive or synergistic, when the outcome is usually significantly greater than the additive response (Tapia de Daza et al., 1996; Leistner, 2000; Ross et al., 2003), because of the disturbing action on microbial homeostasis in several respects (Leistner, 2000).

Because of its great potential for microbial inactivation, several authors have studied the effects of HPH or UHPH, when applied individually or in combination with other mild physical or chemical stresses (heat and H_2_O_2_), on the inactivation of *Bacillus* and *Clostridium* spores, whose genera, from a safety point of view, the most important species belong. The analysis of the literature shows that the major application for HPH and UHPH regards the inactivation of spores of spoiling bacteria while the reports dealing with the inactivation of spores from pathogenic species are sporadic. Focusing on pathogenic species, Chaves-López et al. (2009) evaluated the influence of HPH treatment, applied individually (one, two, or three cycles) or in combination with other mild physical or chemical stresses (mild heat treatment, H_2_O_2_, and low pH), on the capability of *B. cereus* and *B. subtilis* spore, suspended in sterilized double distilled water, to form colonies. These Author determined also the effects of the treatments applied on the release of DPA from the spores, since spore resistance to stresses such as temperature and pressure has been correlated to their ability to retain DPA, present in the core region of the dormant spores (Setlow, 2000; Cortezzo et al., 2004). The Authors outlined how the application of specific stress sequences can significantly inactivate *B. cereus* spores. The remarkable efficacy of repeated cycles at 150 MPa suggested that dynamic high pressure, particularly applied in combination with other sub-lethal stresses, could be a useful and innovative tool for *B. cereus* control in fluid foods. In particular, the Authors showed that, although plate count only slightly decreased in all the strains when one cycle of HPH at 150 MPa was applied alone, the spores released significant levels of DPA (up to 28%) that could indicate a possible disruption of spore layers. Three consecutive cycles of HPH determined high reduction of colony count (about 5 log CFU/ml) and high DPA release (52%). Among the stress conditions applied, it was observed that only the thermal shock after one HPH cycle reduced the colony count of 2.3 log CFU/ml and induced a DPA release up to 57%. Pinho et al. (2011) evaluated the inactivation of *Clostridium sporogenes* PA 3679 spores (considered as harmless twin of *C. botulinum*) by HPH in model system such as skim milk, showing that that pressures up to 300 MPa were not able to cause any reduction on spore counts or promote changes on their thermal resistance. The application of heat shock (100°C/15 min) before HPH treatment and the homogenization process realized at mild inlet temperature (45°C), which results in homogenization temperature of around 84°C at 300 MPa, also did not cause reduction on viable spores counts. A few spores reduction (0.67 logarithmic cycles) were only observed when the milk samples were subjected to homogenization treatment for 16 cycles (multiple passes) at 300 MPa. Therefore, although HPH be recognized as an effective method for milk pasteurization, in this specific case, HPH process is not able to guarantee the commercial sterility of milk, being necessary the association of the homogenization with another preservative method, as refrigeration.

Also Amador Espejo et al. (2014) tested the ability of Ultra High-Pressure Homogenization treatments at 300 MPa with inlet temperatures of 55, 65, 75, and 85°C to inactivate *B. cereus* spores inoculated into commercial ultra high temperature treated whole milk in order to evaluate the inactivation level achieved. These Authors provided important evidence of the suitability of UHPH technology for the inactivation of spores in high numbers, leading to the possibility of obtaining commercially sterile milk. In fact, UHPH conditions at 300 MPa with a inlet temperature of 75 and 85°C were capable of a spore inactivation of 5 log CFU/ml. Furthermore, under these processing conditions, commercial sterility (evaluated as the complete inactivation of the inoculated spores) was obtained in milk treated at 300 MPa with inlet temperature of 75°C.

## Potential of High Pressure Homogenization to Increase the Antimicrobial Activity of Enzyme of Food Interest

Because of the great interest within the food industry in aldehydes, ketones, esters as natural antimicrobial compounds (Burt, 2004; Patrignani et al., 2015b) or enzymes such as lysozyme, lactoperoxidase system and lactoferrin, many Authors have tried to find strategies to enhance their efficacy in foods. In general, the approaches used are aimed to destabilize the microbial outer membranes of Gram-negative bacteria or to modify the chemical structure of the enzyme. For example, Ibrahim et al. (1993) and Bernkop-Schnurch et al. (1998) showed that the antimicrobial spectrum of lysozyme can be extended, including also Gram-negative bacteria, by slight chemical modification of the protein with hydrophobic ligands throughtout thermal treatment. These modifications generate an amphitropic protein able to spread the cytoplasmic membrane (Vannini et al., 2004; Iucci et al., 2007). Peptic digestion or heat treatment are reported to augment the antimicrobial activity of lactoferrin. In this perspective, several authors tried to modify the enzyme structure, and consequently its activity, by using HPH in order to increase food safety. On the other hand the effect of HPH on naturally occurring food enzymes involved also in shelf-life, ripening and functionality of several matrices has been demonstrated (Kheadr et al., 2002; Lanciotti et al., 2004b, 2006; Vannini et al., 2008).

From a safety point of view, Vannini et al. (2004) evaluated the effect of HPH on the activity of antimicrobial enzymes such as lysozyme and lactoperoxidase against a selected group of Gram-positive and Gram-negative species inoculated in skim milk, formulating the hypothesis that the interaction of HPH and enzymes is associated to conformational modifications of the two proteins with a consequent enhancement of their activity. *L. monocytogenes* was the most pressure resistant species while *S. typhimurium*, *S. aureus*, and *S. enteritidis* were found to be very sensitive to the hyperbaric treatment. The enzyme addition enhanced the immediate pressure efficacy on almost all the considered species as indicated by their instantaneous viability loss following the treatment. Moreover, the combination of the enzyme and HPH significantly affected the recovery and growth dynamics of the considered species. Although *L. monocytogenes* was slightly sensitive to pressure, the combination of the two stress factors induced a significant viability loss within 3 h and an extension of lag phases in skim milk during incubation at 37°C. Iucci et al. (2007) investigated the effects of HPH treatment at 100 MPa, in comparison to different heat treatments, 70°C for 30 s, 70°C for 5 min or 100°C for 5 min, on the activity of lysozyme and lactoferrin. Their antimicrobial activities were tested on *L. monocytogenes* inoculated in milk or cultural medium. The results indicated that antimicrobial activities of lactoferrin and lysozyme were enhanced and/or accelerated by HPH treatment. Particularly, the highest immediate inactivation values were recorded when *L. monocytogenes* cells were added to HPH-treated lactoferrin, processed simultaneously or separately with the target microorganism. Although to a lesser extent than HPH treatment the heat treatments applied also were able to increase the antimicrobial activity of lysozyme. The Author suggested that the large supramolecular structure is disrupted under pressure, allowing the components to move freely and become independent of the original structure. Interactions can reform when the pressure instantaneously decreases but the original structure is not reformed because of the independent movements of the components.

Patrignani et al. (2013b) found for *S. enteritidis*, inoculated in LWE, HPH inactivation linearly correlated to the number of passes at 100 MPa underling as for LWE, it is important to take into consideration the additional effect of HPH on the antimicrobial activity of naturally occurring enzymes, such as lysozyme. In fact, dynamic pressure is reported to enhance antimicrobial activity of lysozyme and lactoferrin, probably due to the change of the exposure of hydrophobic regions (Iucci et al., 2007). Guerzoni et al. (2002) hypothesized that the inactivation of *S. enteritidis* in egg based formulation treated at different HPH pressure and its inability to re grow in the system at 10°C was due to the increase of activity of HPH treated lysozyme from eggs. Also Velazquez-Estrada et al. (2008) attributed higher inactivation of *S. enterica* serovar *Senftenberg* 775Win HPH LWE to effects of pressure on cells and naturally occurring antimicrobial enzymes. Diels et al. (2005) observed that, above 150 MPa, *E. coli* became more sensitive to lysozyme when this compound was added before HPH treatment compared with adding the enzyme after the treatments, indicating that HPH treatment increases effectiveness and action spectrum of lysozyme.

## Conclusion

The critical evaluation of the available literature data has showed the great potential of HPH for microbial inactivation and food safety purpose. Since 1990 several Authors have been tested its potentialities *in vitro* and *real* systems, demonstrating its different ability for pathogenic species inactivation in relation to the strains considered, the food matrix and technological procedures adopted. However, until the introduction of new valve design and ultra high pressure homogenizers, able to reach pressures of 400 MPa, this technology was implemented in food industry only for fat globule reduction, for juice treatment and emulsion creation. The introduction of these new variables have opened new field in food sector, also for food decontamination and permitting to replace or minimize the traditional thermal treatments generally applied for the safety purposes. The new main applications regard the treatment of milk (for consumption or dairy product manufacture), fruit and vegetable juice, vegetable milks, and food component (such as enzymes) obtaining more stable and safer products without detrimental effects on quality properties. In fact, in the most of the cases, the literature data have underlined an improvement of the sensory and nutritional properties and stability of the HPH and UHPH treated products. The replacement of traditional thermal treatment can represent an advantage for the industry since this HPH is a cold technology with a lower impact on the environment, more sustainable, saving energy, time and additional costs. Moreover the literature data have demonstrated that HPH, when applied to the milk for cheesemaking, can increase the cheese yield, reduce the cheese hydrolytic patterns, reducing the costs for ripening.

Moreover, another important improvement of the state of the art was done for the inactivation of resistant endospores, which still represent a great challenge for food industry. The literature data have pointed out that the combination of UHPH and several chemico-physical hurdles can be regarded as tool for spore inactivation especially for milk based products. Further improve of food safety and functional properties could be achieved exploiting the well recognized dynamic pressure potential to obtain nanoparticles of antimicrobial molecules or functional ingredients. However, also in this case the reduction of thermal treatment can represent an advantage for food industry and contribute to the maintenance of the quality and nutritional properties of foods.

However, although the achieved aims, UHPH has not been yet implemented in food industry since conventional and ultra homogenizer do not guarantee by themselves the sterilization and, subsequently, packaging of foods in aseptic conditions is needed. This results in a great disadvantage for food industry because it limits the implementation of this technology in food process as full alternative to thermal treatment.

## Author Contributions

FP and RL put in writing this review together consulting the available literature, patents on the topic and trying to investigate several aspects on high pressure homogenization and safety.

## Conflict of Interest Statement

The authors declare that the research was conducted in the absence of any commercial or financial relationships that could be construed as a potential conflict of interest.
